# Visual but Not Auditory-Verbal Feedback Induces Aftereffects Following Adaptation to Virtual Prisms

**DOI:** 10.3389/fnins.2021.658353

**Published:** 2021-10-25

**Authors:** Alexia Bourgeois, Audrey Schmid, Francesco Turri, Armin Schnider, Radek Ptak

**Affiliations:** ^1^Laboratory of Cognitive Neurorehabilitation, Faculty of Medicine, University of Geneva, Geneva, Switzerland; ^2^Division of Neurorehabilitation, Department of Clinical Neurosciences, Geneva University Hospitals, Geneva, Switzerland

**Keywords:** prism adaptation, virtual reality, after-effect, visual, auditory

## Abstract

Visuo-motor adaptation with optical prisms that displace the visual scene (prism adaptation, PA) has been widely used to study visuo-motor plasticity in healthy individuals and to decrease the lateralized bias of brain-damaged patients suffering from spatial neglect. Several factors may influence PA aftereffects, such as the degree of optical deviation (generally measured in dioptres of wedge prisms) or the direction of the prismatic shift (leftward vs. rightward). However, the mechanisms through which aftereffects of adaptation in healthy individuals and in neglect affect performance in tasks probing spatial cognition remain controversial. For example, some studies have reported positive effects of PA on auditory neglect, while other studies failed to obtain any changes of performance even in the visual modality. We here tested a new adaptation method in virtual reality to evaluate how sensory parameters influence PA aftereffects. Visual vs. auditory-verbal feedback of optical deviations were contrasted to assess whether rightward deviations influence manual and perceptual judgments in healthy individuals. Our results revealed that altered visual, but not altered auditory-verbal feedback induces aftereffects following adaptation to virtual prisms after 30-degrees of deviation. These findings refine current models of the mechanisms underlying the cognitive effects of virtual PA in emphasizing the importance of visual vs. auditory-verbal feedback during the adaptation phase on visuospatial judgments. Our study also specifies parameters which influence virtual PA and its aftereffect, such as the sensory modality used for the feedback.

## Introduction

Prismatic adaptation (PA) is a very effective technique to examine short-term sensory-motor plasticity in the healthy or injured brain. Left optical prisms, often used in studies of healthy individuals, displace the entire visual field and induce a compensatory bias in manual reaching or pointing to visual targets (Redding et al., 2005). Once the prisms are removed, pointing movements deviate in the direction opposite the optical shift (the so-called aftereffect). Since an initial report suggested that rightward PA may alleviate signs of spatial neglect (Rossetti et al., 1998) this technique has widely been studied in brain-damaged patients suffering from neglect. Some studies suggest that PA may be effective in the short term and after a single adaptation session (corresponding to 50–60 pointing movements), while repeated sessions may be necessary to induce long-lasting improvements (Frassinetti et al., 2002; Luaute et al., 2006b; Serino et al., 2007, 2009). These early findings suggested a simple and effective method to treat attentional disorders that produces a severe handicap in many activities of daily life. Unfortunately, not all neglect patients respond to the treatment, and several clinical trials failed to provide evidence for lasting effects of PA on activities of daily life or even routine clinical measures of neglect (Luaute et al., 2006a; Rousseaux et al., 2006; Nys et al., 2008; Turton et al., 2010; Barrett et al., 2012; Rode et al., 2015; Lunven et al., 2019; Vilimovsky et al., 2021). Several hypotheses have been explored to explain the absence of PA benefits, for example that damage to brain regions crucially involved in spatial neglect also impairs recalibration following PA (Pisella et al., 2006; Chapman et al., 2010; Kuper et al., 2014), the presence of motor-intentional neglect (Goedert et al., 2014) or differences in adaptation techniques (Ladavas et al., 2011). However, no single explanation seems sufficient to explain all inconsistencies between PA aftereffects in healthy individuals and in neglect patients (Ptak and Schnider, 2020; Qiu et al., 2021).

Several parameters may modulate adaptation effects in healthy subjects, such as the presence or absence of a visual feedback (Freedman, 1968), movement speed (Kitazawa et al., 1997; Redding et al., 2005 for a review), the degree of optical deviation (Barrett et al., 2012; Michel and Cruz, 2015), or the modality of the target to which participants point during the adaptation stage (Calzolari et al., 2017). The latter effect is of particular interest to elucidate whether PA is particularly linked to a visual reference frame or affects spatial frames irrespective of the sensory modality. Interestingly, Sarlegna et al. (2007) showed that visual, kinesthetic, or verbal feedback could be used to drive sensorimotor adaptation, highlighting that adaptation is a multisensory process whose efficiency does not depend on the sensory channel conveying the error signal. Schmitz and Bock (2014) tested adaptation to auditory targets while providing indirect feedback about performance (lateralized sound indicating to move further to the left or right) and found that although adaptation was slower in the auditory than the visual modality, all groups eventually reached asymptote indicating a similar degree of adaptation. Interestingly, this study revealed larger transfer of adaptation effects from the visual to the auditory modality than in the inverse direction. In a later study the same authors observed similar aftereffects of adaptation to visual or auditory targets in pointing, even if subjects simultaneously adapted with each arm to a different modality (Schmitz and Bock, 2017). In a study by Calzolari et al. (2017) subjects wore wedge prisms displacing the visual field rightward by 11.4° while performing pointing movements to visual, auditory, or audio-visual targets. Pointing to auditory or audio-visual targets induced adaptation effects in straight-ahead pointing that were comparable across modalities, suggesting crossmodal integration of different input modalities during prism exposure. This finding is complementary to the observation that rightward prismatic adaptation may alleviate some auditory symptoms in neglect. Tissieres et al. (2017) investigated the effect of rightward PA in patients with auditory neglect and found a beneficial effect of prism exposure on left ear extinction in dichotic listening (see also Eramudugolla et al., 2010; Jacquin-Courtois et al., 2010). Together, these results suggest that PA may affect multisensory, rather than exclusively visual representations of space.

One question emerging from these findings is whether these sensorimotor adaptation effects are driven by the modality of the error signal during adaptation, or the spatial position of the pointing target irrespective of its modality. In the study by Calzolari et al. (2017) subjects performed pointing movements to a sound source, and the error signal was provided by the mismatch between the visual image of their hand and the spatial position of the sound. It is possible that under this setting, the sound provides similar information about spatial position as a visual target does. Indeed, error-based learning has been identified as the driving force of visuomotor adaptations (Wolpert et al., 2011). However, contrary to visuomotor rotation paradigms (see e.g., Shadmehr et al., 2010; Wolpert et al., 2011; Kim et al., 2021), in the visual condition of our study, the error signal was provided in the form of a slight mismatch between visual feedback (position of the controller) and internal feedback (efference copy)/proprioceptive feedback (position of the hand). In this condition subjects always had a visual control about their pointing precision. In the auditory-verbal condition, feedback was provided at the end of the movement in form of a verbal command. An alternative approach is to present during the adaptation phase an auditory error signal regarding the hand position, and thus to perform an adaptation fully in the auditory modality. Influencing spatial reference frames through auditory feedback error would provide a possible alternative to visual prisms that might further be explored in clinical studies on spatial neglect.

In the present study, we compared the effect of visual vs. auditory-verbal feedback during the adaptation phase on visuospatial judgments. We took advantage of the recent development of an alternative adaptation technique that uses virtual reality, rather than classic wedge prisms to induce PA effects (Gammeri et al., 2020; Bourgeois et al., 2021). Whereas wedge prisms introduced a mismatch between the sight of the hand and the real hand, and a mismatch between the sight of the target and the real target, virtual PA introduced a mismatch between the sight of a controller that subjects hold in their hand and the visual pointing target. Research using virtual adaptation therapy is scarce as this is a new area of interest. One study compared the effect of virtual PA with conventional PA (Ramos et al., 2019). The authors found that simulated prism exposure in immersed virtual reality produced larger prismatic after-effects than conventional PA, which calls for further use of virtual PA in adaptation therapy. Indeed, this technique has two important advantages relative to optical prisms. First, any amount of mismatch can be introduced simply by modifying the horizontal position of the controller. While wedge prisms are generally limited to 15–20 dioptres, virtual prisms can deviate by 30 or more degrees, without any optical deformation or artifact. Second, the mismatch can be introduced gradually across several pointing trials, which makes it difficult to detect by the subject or patient. Previous studies have shown that a progressive, multiple-step unaware condition was associated with larger negative after-effects and greater robustness compared to a single-step procedure (aware condition) (Michel et al., 2007). Moreover, previous studies of motor control have shown that subjects adapt to and retain gradually introduced perturbations similarly or even better than sudden perturbations, whether these affect kinematic or dynamic properties of the movement (Kagerer et al., 1997; Klassen et al., 2005).

Using this technique, we previously observed adaptation effects in healthy participants that were proportional to the spatial mismatch, as well as transfer effects on line bisection with the highest adaptation error (30°; Gammeri et al., 2020). Importantly, in contrast to most studies using wedge prisms these findings were obtained with a rightward deviation, which does generally not produce significant transfer of PA in healthy participants. Based on these findings we here replicated the virtual PA effects in healthy participants and additionally tested whether significant PA effects can be induced through auditory-verbal feedback. The main hypothesis of our study was that if sensorimotor adaptation effects are driven by the spatial position of the pointing target, both visual and auditory-verbal feedback should induce adaptation effects.

## Materials and Methods

### Participants

Fourty-eight healthy volunteers (27 women, mean age 26, range 18–46 years) with normal or corrected to normal vision and no history of neurological or psychiatric disorders participated in this study. Participants were all right-handers based on scores of the Edinburgh Handedness Inventory (Oldfield, 1971). They were randomly assigned to one of four groups (12 participants per group) defined by the degree of optical deviation (0 degree of deviation; 30 degrees of deviation) and sensory modality (visual, auditory-verbal) used for the induction of adaptation effects. They were neither informed about group affiliation nor that an optical deviation is induced in some participants. All participants gave informed consent, according to procedures approved by the local ethical committee (University of Geneva, Switzerland).

### Apparatus, Stimuli, and Procedure

**FIGURE 1:**
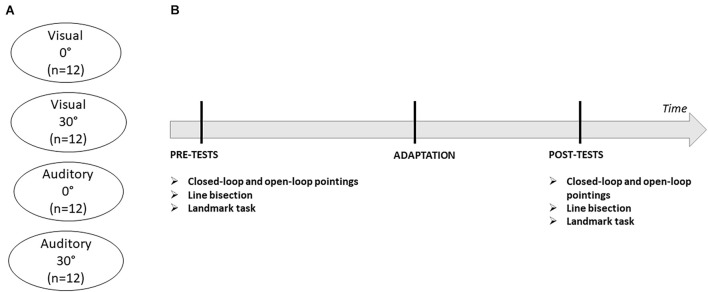
**(A)** Description of the four experimental groups. **(B)** Timeline of the experiment. Each experimental session consisted of three phases (pre-tests, adaptation, and post-tests). Pre- and post-test phases evaluated the presence of adaptation effects on visual closed-loop and visual open-loop pointings and their transfer to line bisection and landmark tasks.

The experiment was programmed using Unity 3D software. A Vive VR system (HTC Corp., Taoyuan, Taiwan) was used to present all stimuli. Participants sat in a chair while wearing the VR headset and responded with the help of a controller held in their right hand whose position was tracked in real-time. The VR headset had a field of view of 110 degrees at a refresh rate of 90 Hz. The spatial resolution of the VR system was less than a millimeter. Before each experimental task, the VR-system was calibrated in order to align the origin of the 3D virtual space to the midline of the subject’s trunk. Head alignment was also monitored on-line through all phases and corrected during the experiment if any head movements with a translation or a rotation of more than 5 mm occurred. Each subject participated to one experimental session, consisting in a baseline test phase, the adaptation phase and a series of post-tests. The baseline and post-test phases were composed of tests designed to evaluate the presence of adaptation effects (visual closed-loop pointing and visual open-loop pointing) and the transfer of adaptation effects to bisection judgments (line bisection and landmark task). The order of these four tasks was randomized and counterbalanced across participants. At the end of the experimental session, participants provided oral feedback about their subjective experience in order to probe whether they had guessed an association between pointing performance and target deviation during the adaptation phase (Figure 1).

#### Adaptation

Participants saw a feature-less white space through the VR headset. The image of the controller was replaced by the image of a white rod, moving coherently with hand movements. An adaptation trial started with the presentation of a black sphere in front of the participant (2.6 degrees of visual angle), approximately at chest height and at arm length. At the beginning of each trial, the sphere turned red and participants were required to reach it with the white rod. As soon as the rod came into contact with the sphere the latter turned black, and participants returned their arm to the start position. Unbeknownst to the participants the perceived position of the controller (the white rod) was gradually shifted rightward relative to its real position, inducing participants to point more to the left to compensate for this bias. PA was thus induced by creating a mismatch between the true position of the target and the subject’s hand (a demonstration of this manipulation can be seen in Supplementary Videos “Adaptation”). The adaptation started after the 15th trials to match the number of visual offsets at the beginning of the experiment between the Visual and the Auditory-verbal condition. The rightward deviation was induced in very small steps (between 0.15 and 0.5 degrees per trial) so that the maximal deviation (30 degrees) was reached after 120 s, which corresponds to approximately 50–70 trials, depending on individual pointing speed. Each participant then continued to point at the maximal deviation until reaching 100 pointing trials. The main reason for applying a gradual adaptation was to conceal the perturbation from participants.

The auditory-verbal condition started in the same way as visual adaptation, where the target and the controller were both visible for the first 8 trials. The target then became invisible while participants continued to reach in its direction with the controller (white rod) still visible. At this stage they heard a voice through the headphones that gave feedback about their pointing performance. If the controller hit the invisible target the word “Correct” was presented, while when it deviated from target the feedback “More to the right” or “More to the left” was given. This auditory feedback was expected to induce a correction of the pointing movement in the next trial. After 15 trials the controller became invisible and subjects continued to execute pointing movements in a completely blank space, hence basing their pointing movements on the real or displaced position of the target according to the auditory feedback. In the 30° deviation condition, the position of the controller gradually shifted to the right in the same way as in the visual adaptation procedure. Correspondingly subjects more frequently heard the feedback “More to the left” which invited them to correct their next arm movement by pointing more to the left. Participants made pointing movements until the full deviation of 30° was reached (after 50–70 trials) and then made further movements until reaching 100 trials.

#### Visual Closed-Loop and Visual Open-Loop Pointings

Visual closed-loop and open-loop pointings were tested with the VR system in a monotonous white visual environment. The VR-system was calibrated in order to align the origin of the 3D virtual space to the midline of the subject’s trunk before each task and monitored on-line during the whole experiment.

Visual open-loop pointing corresponded to straight-ahead pointing, where subjects were required to point straight ahead at a position corresponding to their mid-sagittal line. In the visual closed-loop pointing, a black dot (2.6 degrees of visual angle) was projected exactly in front of the participants and centered on their sagittal midline. In both conditions the controller was not visible and pointing was therefore performed under proprioceptive guidance. Note that the term “open-loop” is generally used in the literature on PA to designate absence of visual feedback of the arm (even though proprioceptive feedback is still available).

Participants held the controller at chest height (start position), reached to the visible target or an imagined point lying exactly straight ahead, and pressed the controller button to record its end position. They performed five trials for each pointing tasks.

#### Line Bisection

Participants were asked to bisect a series of black lines projected on a white background (thickness: 1.15 degrees of visual angle), whose horizontal extent covered 35, 50, or 65 degrees of visual angle. The controller was visible and projected a red light from its top end, allowing participants to bisect the line at its estimated center. After each trial participants were required to return to the start position (holding the controller at chest height). There were three trials for each line length, presented in random order.

#### Landmark Task

In this task, participants were asked to judge whether a red vertical mark (11.4 degrees length) was placed exactly at a position corresponding to their mid-sagittal line or displaced by 3, 6, or 9% to the right or to the left of the center. The mark was presented centrally in 6 trials and displaced leftward in 9 trials (3 trials per displacement) and rightward in another 9 trials, resulting in a total of 24 trials. Participants were asked to orally indicate whether the vertical mark was centered on their sagittal midline or displaced leftward or rightward. In order to assess the subjective center, we computed a subjective bias by assigning a score assessing the distance between the deviation from the objective mark and the response given by the participants. For instance, for a landmark presented left of center, a participant would receive values of 0 for the answer “leftward displaced,” + 1 for the answer “centered” and + 2 for the answer “rightward displaced.” The subjective bias score corresponded to the average of all scores, by excluding trials in which the line was bisected in its center. Thus, an overall positive bias indicated that the subjective center was shifted to the right, while a negative bias indicated a leftward shift.

#### Awareness Questionnaire

An awareness questionnaire was used to evaluate the degree of awareness of the spatial deviation between controller and targets during the adaptation phase. The questionnaire started with a general open question probing any awareness of the visual shift (“Did you feel something strange when you were asked to reach toward the red spheres?”). Participants then answered progressively more concrete questions about their subjective experience (e.g., “I felt that the black dot was not in front of me”) by providing ratings on a five-point Likert scale ranging from “strongly agree” to “strongly disagree.”

## Results

### Visual Closed-Loop and Visual Open-Loop Pointings

In order to evaluate PA aftereffects, repeated-measures ANOVA for the visual closed-loop and open-loop pointings were performed with Time (pre-PA, post-PA) as within-subjects factor and the four experimental groups (auditory-verbal 0°, auditory-verbal 30°, visual 0°, visual 30°) as categorical factor.

**FIGURE 2:**
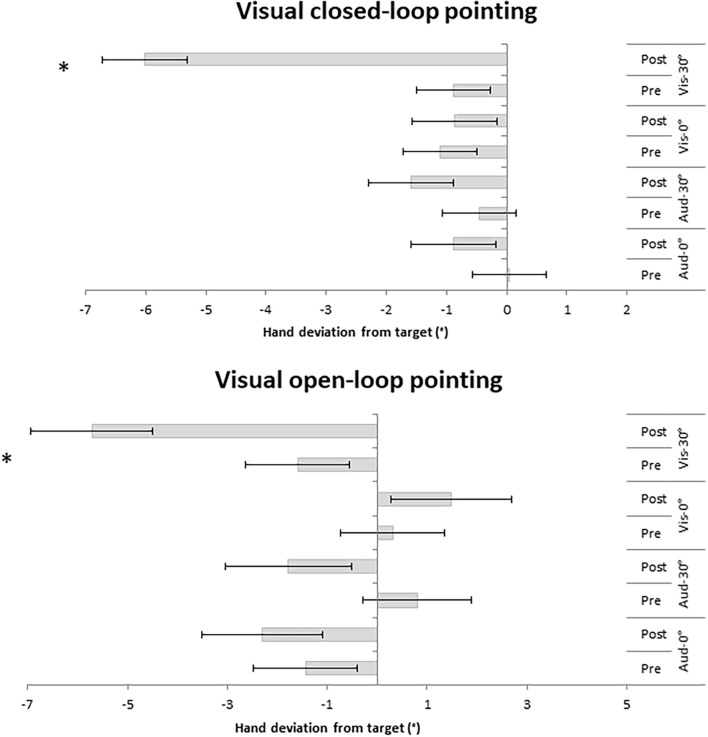
Hand deviation from target (in degree) measured in the visual closed-loop and visual open-loop pointings pre- and post-PA. Negative values indicate leftward deviation, while positive values indicate rightward deviation. Error bars represented standard errors. ^∗^Denotes significant comparisons (*p* > 0.05).

For the visual closed-loop pointing, the analysis indicated main effects of Time [*F*_(1,44)_ = 36.54, MSE = 1.98, *p* < 0.001; η^2^ = 0.45] and Group [*F*_(3,44)_ = 5.20, MSE = 8.45, *p* = 0.004; η^2^ = 0.26], as well as a significant interaction between Time and Group [*F*_(3,44)_ = 16.63, MSE = 32.91, *p* < 0.001; η^2^ = 0.53]. Bonferroni *post hoc* tests of pre- and post-PA in each group revealed PA aftereffects in the Visual 30° group (*p* < 0.001), but not in the other groups (all *p* = 1; Figure 2).

For the visual open-loop pointing, one participant of the group Auditory-verbal 30° was discarded because of an exceedingly high pre-PA hand deviation (> 2 SD from the participants’ mean). The analysis yielded main effects of Time [*F*_(1,43)_ = 16.85, MSE = 3.56, *p* < 0.001; η^2^ = 0.28] and Group [*F*_(3,43)_ = 3.35, MSE = 26.99, *p* = 0.028; η^2^ = 0.19], as well as a significant interaction between Group and Time [*F*_(3,43)_ = 8.69, MSE = 3.56, *p* < 0.001; η^2^ = 0.38]. *Post hoc* tests revealed significant aftereffects for the Visual 30° group (*p* = 0.005), but not in the other groups (Visual 0°: *p* = 1; Auditory-verbal 30°: *p* = 0.070; Auditory-verbal 0°: *p* = 1; Figure 2).

**FIGURE 3:**
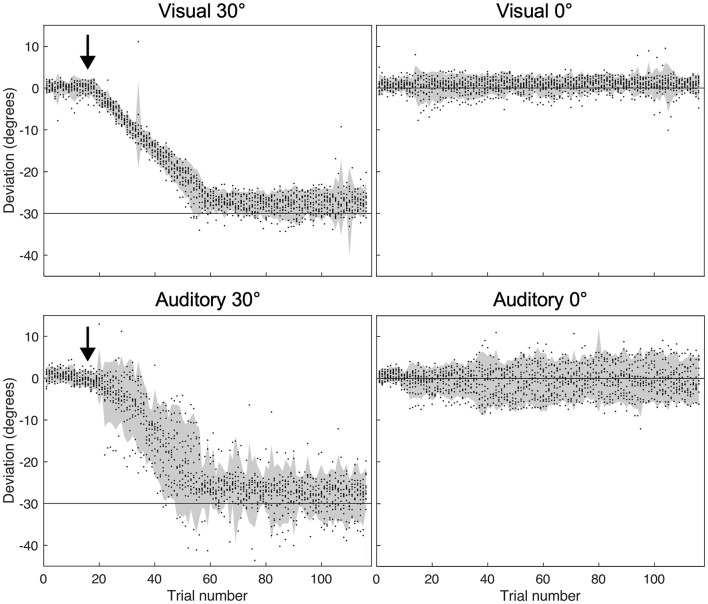
Hand deviation from target measured before and during the adaptation phase (start of adaptation indicated by the black arrow). The gray area represents the 95th confidence interval for each group, and the black dots show deviations of individual subjects in every trial. Negative values indicate leftward deviation, while positive values indicate rightward deviation.

Since no significant adaptation effects were observed in the auditory-verbal condition, we wondered whether the instruction to go “further to the left” was effective in inducing any hand deviation *during* adaptation in the Auditory-verbal 30° group. The controller position was recorded throughout the adaptation phase for the four experimental groups in order to ensure that a deviation was well observed in the condition with 30 degrees of deviation. As shown in Figure 3, participants similarly deviated leftward in the Visual 30° and the Auditory-verbal 30° group. Though the latter group exhibited greater variability of pointing movements, this can be explained by the generally lower precision of pointing movements under auditory guidance (as can also be seen when comparing Auditory-verbal 0° with the Visual 0°conditions).

### Line Bisection

**FIGURE 4:**
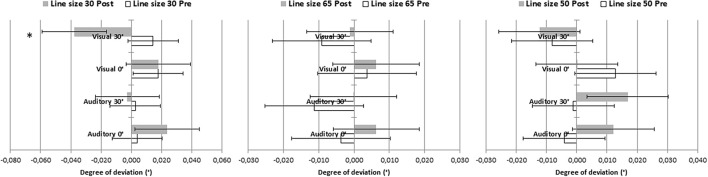
Degree of deviation obtained on the line bisection for each line size (35, 50, 65 degrees of visual angle) pre- and post-PA. Negative values indicate leftward deviation, while positive values indicate rightward deviation. Error bars represented standard errors. ^∗^Denotes significant comparisons (*p* > 0.05).

Since line length is known to affect bisection judgments, we analyzed the line bisection data with a repeated-measures ANOVA with Time (pre-PA, post-PA) and Line size (35, 50, 65 degrees of visual angle) as within-subject factors and Group as categorical factor (Figure 4). The analysis yielded a significant interaction between Time, Line size and Group [*F*_(6,88)_ = 3.21, MSE = 0.001, *p* = 0.007; η^2^ = 0.18]. Bonferroni-comparisons revealed a significantly larger rightward deviation post-PA as compared to pre-PA in the Visual 30° group, but only for the shortest line corresponding to 35° of visual angle (*p* < 0.001). No other effect was significant.

### Landmark Task

Table 1 shows the degree of subjective bias in the landmark task pre- and post-PA. Since these data were not interval-scaled, we compared pre- and post-PA subjective bias scores for each group using Wilcoxon tests. These analyses yielded no significant differences (all p*s* > 0.093).

**TABLE 1:** Mean subjective bias score for each condition.

	**Visual 0°**	**Visual 30°**	**Auditory-verbal 0°**	**Auditory-verbal 30°**
Pre-PA	−0.31	−0.28	−1.35	−0.36
Post-PA	−0.09	−0.31	−0.79	−0.19

### Awareness Questionnaire

Only four subjects (3 subjects in the Visual 30° group and 1 subject in the Visual 0° group) reported that they had perceived a visual shift between the controller and pointing targets during the adaptation phase as probed with the Awareness questionnaire. The number of subjects was thus too low to probe related-effects of awareness on prismatic adaptation.

## Discussion

Previous studies have repeatedly demonstrated robust aftereffects of PA in healthy individuals during pointing toward visual targets (Colent et al., 2000; Michel et al., 2003; Schintu et al., 2014; Michel, 2015). Adaptation effects have been observed when a visual feedback about arm/hand position was given (closed loop) but also when this feedback was absent (open loop). Based on these observations, one might hypothesize that adaptation effects are not linked to a sensory modality but rather depend on a modality-independent spatial reference system. Thus, we here tested whether adaptation effects might be induced through auditory-verbal feedback in inducing a mismatch between hand and target position, similarly to the visual mismatch induced by deviating prisms. We examined this question using a virtual reality adaptation method, which avoids blindfolding participants for the auditory adaptation. Our results revealed that visual, but not auditory-verbal feedback induces aftereffects following adaptation to virtual prisms after 30-degrees of deviation.

### Adaptation Effects in the Auditory Modality

Only few studies investigated the contribution of the auditory modality to prism adaptation effects (Eramudugolla et al., 2010; Jacquin-Courtois et al., 2010; Calzolari et al., 2017; Michel et al., 2019; Matsuo et al., 2020). Jacquin-Courtois et al. (2010) demonstrated that PA can improve left auditory extinction in visual neglect patients, indicating that PA may interact with higher-order brain functions related to multisensory integration. Also, fMRI studies reported similar fronto-parietal activation for visual and auditory stimuli (Smith et al., 2010), suggesting that visual and auditory attention may rely on a supramodal attentional network. Of particular relevance for our findings is the study by Calzolari et al. (2017), who observed adaptation effects with right-deviating prisms when participants pointed toward visual, auditory, or audio-visual targets. During adaptation, subjects shifted their pointing movements leftward irrespective of the target modality. In addition, aftereffects measured with straight-ahead pointing were comparable between all three adaptation conditions, suggesting a supramodal effect of prisms. The main difference with our study is that these authors used different target modalities (e.g., a sound played through a loudspeaker located ahead of the participant) but kept the adaptation procedure constant (pointing with eyes open). Participants could not see their arm except for the very last part of the pointing movement. However, they knew precisely where their arm was located with respect to the target at the end of each pointing movement. They thus had some visual control about their arm position in all three target conditions. In contrast, our subjects never had any knowledge of their hand position and had to rely completely on the auditory-verbal feedback.

One could argue that the lack of adaptation effects is due to subjects not following the verbal instruction to move more to the left, but as Figure 3 shows this concern can be excluded. All subjects corrected their trajectory across pointing movements and finished the adaptation session with a constant leftward pointing bias. Thus, the lack of adaptation effects measured in the visual closed-loop and open-loop pointings must be due to the fact that simply pointing more to the left (without a visual feedback of the mismatch) may not impact the coordinate system of the peripersonal action space with respect to the body center. This interpretation may also be compatible with the adaptation effects to auditory targets observed by Calzolari et al. (2017) because even with purely auditory stimulation, participants may be able to create a visual “tag” marking the spatial coordinates of the origin of the sound. This visual tag may then play the role of a visual target, that could signal a spatial mismatch in pointing.

A limiting factor of our study is that the auditory-verbal condition possibly provided less (or less precise) spatial feedback about hand and target position. Indeed, as seen in Figure 3 subjects’ responses appear to be more variable when feedback is auditory (in both, the 30 and the 0 degrees conditions). However, the figure also shows that subjects gradually shifted their pointing movements leftward, and ended up with a deviation that was comparable to the visual condition. The higher variability in the auditory-verbal condition can be explained by lower precision of pointing movements under auditory guidance (as is also seen when comparing the Visual 0° and Auditory 0° groups; see also Schmitz and Bock, 2017). A previous study by Schmitz and Bock (2014) used a similar auditory feedback signal as we did and observed adaptation aftereffects in the auditory modality. Increased variability therefore doesn’t seem sufficient to explain the complete absence of adaptation effects in our study.

### The Nature of Error Signals Necessary for Adaptation

A vast amount of literature is concerned with adaptation effects in motor control and the nature of error signals underlying these effects. Sensory-motor adaptation plays a prominent role in the elaboration of models of motor control, as it reflects the plasticity of the motor system and hence its capacity to improve performance across repeated trials (Wolpert and Ghahramani, 2000; Bock, 2013). Computational models posit that the brain uses a forward model of the sensory consequences of a motor command and compares them in true time with the actual sensory outcomes (Shadmehr and Krakauer, 2008; Wong et al., 2015; Haar and Donchin, 2020). Consequently, sensory-motor adaptation might be triggered by the need to reduce an error signal (i.e., a mismatch between a predicted state and the true state, or sensory prediction error) (Shadmehr et al., 2010). In the visual condition of our study, the error signal was provided in the form of a slight mismatch between visual feedback (position of the controller) and internal feedback (efference copy)/proprioceptive feedback (position of the hand). In this condition subjects always had a visual control about their pointing precision. In the auditory-verbal condition, feedback was provided at the end of the movement in form of a verbal command. One may ask whether the absence of adaptation effects in the visual closed-loop and open-loop pointings can be explained by our use of a symbolic, rather than an analogical error signal. However, this seems unlikely, as other study found that a verbal feedback could be used to drive sensorimotor adaptation (Sarlegna et al., 2007), highlighting that adaptation may be a multisensory processing, whose efficiency did not depend on the sensory channel conveying the error signal. Moreover, Schmitz and Bock (2014) found robust adaptation effects when subjects were pointing toward auditory targets and received lateralized sounds indicating to move further to the left or right on the next trial. The major contrast between Schmitz and Bock (2014) and our study is that their participants performed pointing movements toward sounds played through headphones and thus only had to deal with the error signal about their hand position. In contrast, our subjects had to create a representation of target position within the first 15 trials, and then maintain this representation in mind while attempting to correct their pointing movements. While they managed to follow the feedback and to shift their hand gradually leftward, we hypothesize that the simultaneous maintenance of target position and integration of the error signal interfered with lasting recalibration of the motor system. Finally, if significant after-effects are observed for the visual open-loop pointing after 30° of adaptation in the visual modality, a trend seemed to be also observed in the auditory-verbal group after 30° of deviation. Further testing with more subjects could be helpful to definitively clarify this issue.

### Transfer Effects of Adaptation

In addition to adaptation effects in the visual modality, we also observed perceptual transfer effects following visual (but not auditory-verbal) feedback. Interestingly, these effects were restricted to the line bisection task and only to the shortest lines. This result might be explained by differences on exploration strategy. Indeed, we used lines that were much longer than those generally used for paper-and-pencil tasks. In the VR environment, longer lines required eye-movements to be perceived entirely, which may have interfered with or even canceled the adaptation effects by recalibrating the coordinates of the sensory-motor reference frame. We also found no transfer effects in the landmark task, which is in good agreement with previous reports suggesting that PA may affect visuomotor performance more than purely perceptual measure (Harvey et al., 1995; Gammeri et al., 2020; but see Ptak, 2017; McIntosh et al., 2019).

It should be noted that the adaptation and transfer effects we measured were observed with *rightward* deviating prisms, similarly to two previous studies using the same methodology in healthy individuals (Gammeri et al., 2020) and in brain-damaged patients suffering from neglect (Bourgeois et al., 2021). This result contrasts with the results found in healthy individuals with wedge prisms. Indeed, the literature consistently reports an effect of left PA in healthy subjects, whereas right PA does not induce significant behavioral effects. Surprisingly, rightward deviating prisms induce significant BOLD-signal changes in the inferior parietal cortices (Clarke and Crottaz-Herbette, 2016 for a review; Crottaz-Herbette et al., 2014; Schintu et al., 2020), as well as PA-related electrophysiological markers (Martin-Arevalo et al., 2016), though these changes are not accompanied by behavioral modifications. In contrast and in line with our results, a recent study using rightward prism-deviation found that prism exposure in VR produces larger prismatic aftereffects than standard prism exposure in healthy subjects (Ramos et al., 2019). These negative results at a behavioral level using wedge prisms suggest potential limitations of behavioral measures in healthy subjects. One limitation could be the maximal deviating power of optical prisms, which is limited to 20° for prismatic goggles, beyond which a significant discomfort and visual distortion is induced. This maximal deviation may not be enough to induce significant behavioral changes in healthy subjects after rightward deviation. In contrast, the deviation of virtual prisms is only limited by bodily biomechanical constraints, not the VR technology. Another advantage is the possibility to induce the mismatch between hand and target progressively (increasing it from trial to trial), which significantly limits conscious perception of any visual bias and thus eliminates the use of conscious (Michel et al., 2007).

In sum, the findings of this study support the view that sensory-motor adaptation effects can be induced with virtual prisms (as with wedge prisms), and that these effects operate in a visually aligned coordinate system. Our results refine current models of the mechanisms underlying virtual PA’s cognitive effects, as well as parameters which influence PA and its aftereffect.

## Data Availability Statement

The original contributions presented in the study are included in the article/Supplementary Material, further inquiries can be directed to the corresponding author/s.

## Ethics Statement

The studies involving human participants were reviewed and approved by the University of Geneva, Switzerland. The patients/participants provided their written informed consent to participate in this study.

## Author Contributions

AB, FT, RP, and ArS contributed to the design and implementation of the research. AuS collected the data. AB, FT, and RP performed the analysis. AB, RP, and ArS wrote the manuscript. All authors contributed to the article and approved the submitted version.

## Conflict of Interest

The authors declare that the research was conducted in the absence of any commercial or financial relationships that could be construed as a potential conflict of interest.

## Publisher’s Note

All claims expressed in this article are solely those of the authors and do not necessarily represent those of their affiliated organizations, or those of the publisher, the editors and the reviewers. Any product that may be evaluated in this article, or claim that may be made by its manufacturer, is not guaranteed or endorsed by the publisher.

## Supplementary Material

Supplementary Video 1 Demonstration of the adaptation procedure in the visual modality. PA was induced by creating a mismatch between the true position of the target and the subject’s hand.

Supplementary Video 2 Pre- and post-tests: demonstration of the open loop pointing (OPL) with a visual feedback. Participants held the controller at chest height (start position), reached to the visible target or an imagined point lying exactly straight ahead, and pressed the controller button to record its end position.

Supplementary Video 3 Pre- and post-tests: demonstration of the line bisection task.

## References

[B1] Barrett A. M. Goedert K. M. Basso J. C. (2012). Prism adaptation for spatial neglect after stroke: translational practice gaps. *Nat. Rev. Neurol.* 8 567–577. 10.1038/nrneurol.2012.170 22926312 PMC3566983

[B2] Bock O. (2013). Basic principles of sensorimotor adaptation to different distortions with different effectors and movement types: a review and synthesis of behavioral findings. *Front. Hum. Neurosci.* 7:81. 10.3389/fnhum.2013.00081 23503204 PMC3596763

[B3] Bourgeois A. Turri F. Schnider A. Ptak R. (2021). Virtual prism adaptation for spatial neglect: A double-blind study. *Neuropsychol. Rehabil.* 2021 1–15. 10.1080/09602011.2020.1864412 33406997

[B4] Calzolari E. Albini F. Bolognini N. Vallar G. (2017). Multisensory and Modality-Specific Influences on Adaptation to Optical Prisms. *Front. Hum. Neurosci.* 11:568. 10.3389/fnhum.2017.00568 29213233 PMC5702769

[B5] Chapman H. L. Eramudugolla R. Gavrilescu M. Strudwick M. W. Loftus A. Cunnington R. (2010). Neural mechanisms underlying spatial realignment during adaptation to optical wedge prisms. *Neuropsychologia* 48 2595–2601. 10.1016/j.neuropsychologia.2010.05.006 20457170

[B6] Clarke S. Crottaz-Herbette S. (2016). Modulation of visual attention by prismatic adaptation. *Neuropsychologia* 92 31–41. 10.1016/j.neuropsychologia.2016.06.022 27342255

[B7] Colent C. Pisella L. Bernieri C. Rode G. Rossetti Y. (2000). Cognitive bias induced by visuo-motor adaptation to prisms: a simulation of unilateral neglect in normal individuals? *NeuroReport* 11 1899–1902. 10884040 10.1097/00001756-200006260-00019

[B8] Crottaz-Herbette S. Fornari E. Clarke S. (2014). Prismatic adaptation changes visuospatial representation in the inferior parietal lobule. *J. Neurosci.* 34 11803–11811. 10.1523/JNEUROSCI.3184-13.2014 25164675 PMC6608412

[B9] Eramudugolla R. Boyce A. Irvine D. R. Mattingley J. B. (2010). Effects of prismatic adaptation on spatial gradients in unilateral neglect: A comparison of visual and auditory target detection with central attentional load. *Neuropsychologia* 48 2681–2692. 10.1016/j.neuropsychologia.2010.05.015 20478321

[B10] Frassinetti F. Angeli V. Meneghello F. Avanzi S. Ladavas E. (2002). Long-lasting amelioration of visuospatial neglect by prism adaptation. *Brain* 125(Pt 3), 608–623. 10.1093/brain/awf056 11872617

[B11] Freedman S. J. (1968). “Perceptual compensation and learning,” in *The Neuropsychology of Spatial Oriented Behavior* ed. Freedman S. J. (Chicago, IL: The Dorsey Press), 63–76.

[B12] Gammeri R. Turri F. Ricci R. Ptak R. (2020). Adaptation to virtual prisms and its relevance for neglect rehabilitation: a single-blind dose-response study with healthy participants. *Neuropsychol. Rehabil.* 30 753–766. 10.1080/09602011.2018.1502672 30040026

[B13] Goedert K. M. Chen P. Boston R. C. Foundas A. L. Barrett A. M. (2014). Presence of motor-intentional aiming deficit predicts functional improvement of spatial neglect with prism adaptation. *Neurorehabilit. Neural Repair* 28 483–493. 10.1177/1545968313516872 PMC4074266 24376064

[B14] Haar S. Donchin O. (2020). A Revised Computational Neuroanatomy for Motor Control. *J. Cogn. Neurosci.* 32 1823–1836. 10.1162/jocn_a_01602 32644882

[B15] Harvey M. Milner A. D. Roberts R. C. (1995). An investigation of hemispatial neglect using the Landmark Task. *Brain Cogn.* 27 59–78. 10.1006/brcg.1995.1004 7748546

[B16] Jacquin-Courtois S. Rode G. Pavani F. O’Shea J. Giard M. H. Boisson D. (2010). Effect of prism adaptation on left dichotic listening deficit in neglect patients: glasses to hear better? *Brain* 133(Pt 3), 895–908. 10.1093/brain/awp327 20110244

[B17] Kagerer F. A. Contreras-Vidal J. L. Stelmach G. E. (1997). Adaptation to gradual as compared with sudden visuo-motor distortions. *Exp. Brain Res.* 115 557–561. 9262212 10.1007/pl00005727

[B18] Kim H. E. Avraham G. Ivry R. B. (2021). The Psychology of Reaching: Action Selection, Movement Implementation, and Sensorimotor Learning. *Annu. Rev. Psychol.* 72 61–95. 10.1146/annurev-psych-010419-051053 32976728 PMC8514106

[B19] Kitazawa S. Kimura T. Uka T. (1997). Prism adaptation of reaching movements: specificity for the velocity of reaching. *J. Neurosci.* 17 1481–1492. 9006989 10.1523/JNEUROSCI.17-04-01481.1997 PMC6793717

[B20] Klassen J. Tong C. Flanagan J. R. (2005). Learning and recall of incremental kinematic and dynamic sensorimotor transformations. *Exp. Brain Res.* 164 250–259. 10.1007/s00221-005-2247-4 15947919

[B21] Kuper M. Wunnemann M. J. Thurling M. Stefanescu R. M. Maderwald S. Elles H. G. (2014). Activation of the cerebellar cortex and the dentate nucleus in a prism adaptation fMRI study. *Hum. Brain Mapp.* 35 1574–1586. 10.1002/hbm.22274 23568448 PMC6869654

[B22] Ladavas E. Bonifazi S. Catena L. Serino A. (2011). Neglect rehabilitation by prism adaptation: different procedures have different impacts. *Neuropsychologia* 49 1136–1145. 10.1016/j.neuropsychologia.2011.01.044 21310165

[B23] Luaute J. Michel C. Rode G. Pisella L. Jacquin-Courtois S. Costes N. (2006b). Functional anatomy of the therapeutic effects of prism adaptation on left neglect. *Neurology* 66 1859–1867. 10.1212/01.wnl.0000219614.33171.01 16801651

[B24] Luaute J. Halligan P. Rode G. Jacquin-Courtois S. Boisson D. (2006a). Prism adaptation first among equals in alleviating left neglect: a review. *Restor. Neurol. Neurosci.* 24 409–418. 17119314

[B25] Lunven M. Rode G. Bourlon C. Duret C. Migliaccio R. Chevrillon E. (2019). Anatomical predictors of successful prism adaptation in chronic visual neglect. *Cortex* 120 629–641. 10.1016/j.cortex.2018.12.004 30621959

[B26] Martin-Arevalo E. Laube I. Koun E. Farne A. Reilly K. T. Pisella L. (2016). Prism Adaptation Alters Electrophysiological Markers of Attentional Processes in the Healthy Brain. *J. Neurosci.* 36 1019–1030. 10.1523/JNEUROSCI.1153-15.2016 26791229 PMC6602003

[B27] Matsuo T. Moriuchi T. Iso N. Hasegawa T. Miyata H. Maruta M. (2020). Effects of prism adaptation on auditory spatial attention in patients with left unilateral spatial neglect: a non-randomized pilot trial. *Int. J. Rehabil. Res.* 43 228–234. 10.1097/MRR.0000000000000413 32776764

[B28] McIntosh R. D. Brown B. M. A. Young L. (2019). Meta-analysis of the visuospatial aftereffects of prism adaptation, with two novel experiments. *Cortex* 111 256–273. 10.1016/j.cortex.2018.11.013 30530268

[B29] Michel C. (2015). Beyond the Sensorimotor Plasticity: Cognitive Expansion of Prism Adaptation in Healthy Individuals. *Front. Psychol.* 6:1979. 10.3389/fpsyg.2015.01979 26779088 PMC4700133

[B30] Michel C. Bonnet C. Podor B. Bard P. Poulin-Charronnat B. (2019). Wearing prisms to hear differently: After-effects of prism adaptation on auditory perception. *Cortex* 115 123–132. 10.1016/j.cortex.2019.01.015 30822612

[B31] Michel C. Cruz R. (2015). Prism adaptation power on spatial cognition: adaptation to different optical deviations in healthy individuals. *Neurosci. Lett.* 590 145–149. 10.1016/j.neulet.2015.02.001 25660233

[B32] Michel C. Pisella L. Halligan P. W. Luaute J. Rode G. Boisson D. (2003). Simulating unilateral neglect in normals using prism adaptation: implications for theory. *Neuropsychologia* 41 25–39. 10.1016/s0028-3932(02)00135-5 12427563

[B33] Michel C. Pisella L. Prablanc C. Rode G. Rossetti Y. (2007). Enhancing visuomotor adaptation by reducing error signals: single-step (aware) versus multiple-step (unaware) exposure to wedge prisms. *J. Cogn. Neurosci.* 19 341–350. 10.1162/jocn.2007.19.2.341 17280521

[B34] Nys G. M. de Haan E. H. Kunneman A. de Kort P. L. Dijkerman H. C. (2008). Acute neglect rehabilitation using repetitive prism adaptation: a randomized placebo-controlled trial. *Restor. Neurol. Neurosci.* 26 1–12. 18431002

[B35] Oldfield R. C. (1971). The assessment and analysis of handedness: the Edinburgh Inventory. *Neuropsychologia* 9 97–113. 5146491 10.1016/0028-3932(71)90067-4

[B36] Pisella L. Rode G. Farne A. Tilikete C. Rossetti Y. (2006). Prism adaptation in the rehabilitation of patients with visuo-spatial cognitive disorders. *Curr. Opin. Neurol.* 19 534–542. 10.1097/WCO.0b013e328010924b 17102690

[B37] Ptak R. (2017). What role for prism adaptation in the rehabilitation of pure neglect dyslexia? *Neurocase* 23 193–200. 10.1080/13554794.2017.1361452 28774229

[B38] Ptak R. Schnider A. (2020). “Neuropsychological rehabilitation of higher cortical functions after brain damage,” in *Oxford Textbook of Neurorehabilitation*, 2nd Edn, eds Dietz V. Ward N. (Oxford: Oxford University Press), 262–271.

[B39] Qiu H. Wang J. Yi W. Yin Z. Wang H. Li J. (2021). Effects of Prism Adaptation on Unilateral Neglect After Stroke: An Updated Meta-Analysis of Randomized Controlled Trials. *Am. J. Phys. Med. Rehabil.* 100 259–265. 10.1097/PHM.0000000000001557 33595938

[B40] Ramos A. A. Horning E. C. Wilms I. L. (2019). Simulated prism exposure in immersed virtual reality produces larger prismatic after-effects than standard prism exposure in healthy subjects. *PLoS One* 14:e0217074. 10.1371/journal.pone.0217074 31125360 PMC6534293

[B41] Redding G. M. Rossetti Y. Wallace B. (2005). Applications of prism adaptation: a tutorial in theory and method. *Neurosci. Biobehav. Rev.* 29 431–444. 10.1016/j.neubiorev.2004.12.004 15820548

[B42] Rode G. Lacour S. Jacquin-Courtois S. Pisella L. Michel C. Revol P. (2015). Long-term sensorimotor and therapeutical effects of a mild regime of prism adaptation in spatial neglect. A double-blind RCT essay. *Ann. Phys. Rehabil. Med.* 58 40–53. 10.1016/j.rehab.2014.10.004 25543183

[B43] Rossetti Y. Rode G. Pisella L. Farnè A. Li L. Boisson D. (1998). Prism adaptation to a rightward optical deviation rehabilitates left hemispatial neglect. *Nature* 395 166–169. 9744273 10.1038/25988

[B44] Rousseaux M. Bernati T. Saj A. Kozlowski O. (2006). Ineffectiveness of prism adaptation on spatial neglect signs. *Stroke* 37 542–543. 10.1161/01.STR.0000198877.09270.e8 16373638

[B45] Sarlegna F. R. Gauthier G. M. Blouin J. (2007). Influence of feedback modality on sensorimotor adaptation: contribution of visual, kinesthetic, and verbal cues. *J. Mot. Behav.* 39 247–258. 10.3200/JMBR.39.4.247-258 17664168

[B46] Schintu S. Freedberg M. Gotts S. J. Cunningham C. A. Alam Z. M. Shomstein S. (2020). Prism Adaptation Modulates Connectivity of the Intraparietal Sulcus with Multiple Brain Networks. *Cereb Cortex* 30 4747–4758. 10.1093/cercor/bhaa032 32313949 PMC7526755

[B47] Schintu S. Pisella L. Jacobs S. Salemme R. Reilly K. T. Farne A. (2014). Prism adaptation in the healthy brain: the shift in line bisection judgments is long lasting and fluctuates. *Neuropsychologia* 53 165–170. 10.1016/j.neuropsychologia.2013.11.013 24291512

[B48] Schmitz G. Bock O. (2014). A comparison of sensorimotor adaptation in the visual and in the auditory modality. *PLoS One* 9:e107834. 10.1371/journal.pone.0107834 25254660 PMC4177875

[B49] Schmitz G. Bock O. L. (2017). Properties of intermodal transfer after dual visuo- and auditory-motor adaptation. *Hum. Mov. Sci.* 55 108–120. 10.1016/j.humov.2017.08.006 28810171

[B50] Serino A. Barbiani M. Rinaldesi M. L. Ladavas E. (2009). Effectiveness of prism adaptation in neglect rehabilitation: a controlled trial study. *Stroke* 40 1392–1398. 10.1161/STROKEAHA.108.530485 19246708

[B51] Serino A. Bonifazi S. Pierfederici L. Ladavas E. (2007). Neglect treatment by prism adaptation: what recovers and for how long. *Neuropsychol. Rehabil.* 17 657–687. 10.1080/09602010601052006 17852762

[B52] Shadmehr R. Krakauer J. W. (2008). A computational neuroanatomy for motor control. *Exp. Brain Res.* 185 359–381. 10.1007/s00221-008-1280-5 18251019 PMC2553854

[B53] Shadmehr R. Smith M. A. Krakauer J. W. (2010). Error correction, sensory prediction, and adaptation in motor control. *Annu Rev. Neurosci.* 33 89–108. 10.1146/annurev-neuro-060909-153135 20367317

[B54] Smith D. V. Davis B. Niu K. Healy E. W. Bonilha L. Fridriksson J. (2010). Spatial attention evokes similar activation patterns for visual and auditory stimuli. *J. Cogn. Neurosci.* 22 347–361. 10.1162/jocn.2009.21241 19400684 PMC2846529

[B55] Tissieres I. Elamly M. Clarke S. Crottaz-Herbette S. (2017). For Better or Worse: The Effect of Prismatic Adaptation on Auditory Neglect. *Neural. Plast* 2017:8721240. 10.1155/2017/8721240 29138699 PMC5613466

[B56] Turton A. J. O’Leary K. Gabb J. Woodward R. Gilchrist I. D. (2010). A single blinded randomised controlled pilot trial of prism adaptation for improving self-care in stroke patients with neglect. *Neuropsychol. Rehabil.* 20 180–196. 10.1080/09602010903040683 19629848

[B57] Vilimovsky T. Chen P. Hoidekrova K. Petioky J. Harsa P. (2021). Prism adaptation treatment to address spatial neglect in an intensive rehabilitation program: A randomized pilot and feasibility trial. *PLoS One* 16:e0245425. 10.1371/journal.pone.0245425 33481828 PMC7822563

[B58] Wolpert D. M. Diedrichsen J. Flanagan J. R. (2011). Principles of sensorimotor learning. *Nat. Rev. Neurosci.* 12 739–751. 10.1038/nrn3112 22033537

[B59] Wolpert D. M. Ghahramani Z. (2000). Computational principles of movement neuroscience. *Nat. Neurosci.* 3(Suppl.), 1212–1217. 10.1038/81497 11127840

[B60] Wong A. L. Haith A. M. Krakauer J. W. (2015). Motor Planning. *Neuroscientist* 21 385–398. 10.1177/1073858414541484 24981338

